# Non-Conventional Peptide Self-Assembly into a Conductive Supramolecular Rope

**DOI:** 10.3390/nano13020333

**Published:** 2023-01-13

**Authors:** Nicola Forlano, Raffaella Bucci, Alessandro Contini, Mariano Venanzi, Ernesto Placidi, Maria Luisa Gelmi, Raffaella Lettieri, Emanuela Gatto

**Affiliations:** 1 Department of Chemical Science and Technologies, University of Rome “Tor Vergata”, Via della Ricerca Scientifica, 00133 Roma, Italy; 2 Department of Pharmaceutical Sciences, University of Milan, Via Venezian 21, 20133 Milan, Italy; 3 Department of Physics, Sapienza University of Rome, P.le Aldo Moro 2, 00185 Rome, Italy

**Keywords:** self-assembly, peptides, peptidomimetics, smart materials, electron transport, supramolecular chemistry, fibrils

## Abstract

Structures composed of alternating α and β amino acids can give rise to peculiar secondary structural motifs, which could self-assemble into complex structures of controlled geometries. This work describes the self-assembly properties of an α,β-peptide, containing three units of *syn* H2-(2-F-Phe)-h-PheGly-OH, able to self-organize on surfaces into a fascinating supramolecular rope. This material was characterized by AFM, electronic conduction and fluorescence measurements. Molecular dynamics simulations showed that this hexapeptide can self-assemble into an antiparallel β-sheet layer, stabilized by intermolecular H-bonds, which, in turn, can self-assemble into many side-by-side layers, due to π-π interactions. As a matter of fact, we demonstrated that in this system, the presence of aromatic residues at the intramolecular interface promoted by the alternation of α,β-amino-acids in the primary sequence, endorses the formation of a super-secondary structure where the aromatic groups are close to each other, conferring to the system good electron conduction properties. This work demonstrates the capability and future potential of designing and fabricating distinctive nanostructures and efficient bioelectronic interfaces based on an α,β-peptide, by controlling structure and interaction processes beyond those obtained with α- or β-peptides alone.

## 1. Introduction

The supramolecular organization of molecules is a potent tool to confer to materials different macroscopic properties [1,2,3,4]. In nature, cellulose, tendon and hair fibres are obtained through the spontaneous process of self-assembly of cellulose, collagen and keratin, respectively. Trying to mimic this, the bottom-up approach is a way that is increasingly considered by scientists to fabricate smart materials at various scales [2,3,4,5,6,7]. This method deals with the rational design of engineered molecular building blocks that undergo a spontaneous predefined self-assembly process via directional, specific, reversible and modulated non-covalent interactions.

Within biopolymers, peptides and proteins are the most versatile building blocks. As a matter of fact, while polysaccharides are composed of identical monomers, proteins may contain up to 20 different amino acid monomers forming a polypeptide chain. The amino acid sequence will define the final properties of the polymer; for example, proteins can be very elastic materials, like the elastin in the skin, or very strong and structural materials, such as the collagen.

In particular, molecular self-assembly of peptide building blocks composed of α-amino acids into ordered supramolecular structures has gained a lot of interest due to the unique properties of the products, such as biocompatibility, good electronic conduction capability, chemical and structural assortment, robustness and ease of large-scale synthesis [7,8,9,10,11,12,13,14]. Over the last years, we have reported several papers concerning the unique self-assembly properties of helical peptides on gold surfaces [15,16,17,18,19,20,21,22,23,24] and in solution [25,26,27,28]. We discovered that a minimal variation in the primary sequence, may greatly influence the peptide secondary structure and thus, the peptide self-assembly and the final supramolecular structure obtained. Within peptide conformational preferences, β-strand structures are well known to give rise to β-sheets, which are renowned to self-assemble into sophisticated organic supramolecular nanostructures [29]. β-sheets consist of β-strands connected laterally by at least two or three backbone hydrogen bonds, forming a generally twisted, pleated sheet. A β-strand is a stretch of polypeptide chain with a backbone in an extended conformation. The supramolecular association of β-sheets has been implicated in the formation of the fibrils and protein aggregates observed in amyloidosis, in particular in Alzheimer’s disease [30]. However, despite the black side of these structures related to this disease, β-sheets peptide self-assembled structures may find applications in many fields, such as for healthcare products (biosensing platforms, bone regenerations, homeostasis) or for optic/energy purposes (Li-ion batteries, photoluminescent materials, artificial photosynthesis) [31].

Interestingly, not only the primary sequence but also the experimental conditions, such as temperature, pH, type of solvent and ionic strength, may influence the fibrils formation [32].

**Figure 1:**
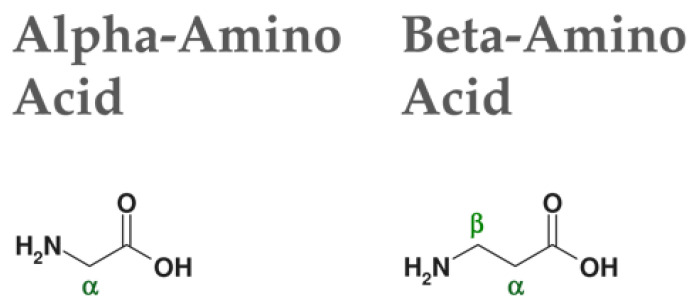
Comparison between α- and β-amino acid.

These versatile and fascinating materials, however, despite being widely studied at the fundamental level, have a problem that strongly limits their industrial use, especially when they are designed to be used as medical devices: they are not resistant to proteolysis. In order to overcome this problem, the use of a novel class of building blocks has been proposed: β-amino acids [33]. β-amino acids differ from α-amino acids in the presence of an additional carbon atom in the amino acid backbone (Figure 1).

This change gives rise to four possible diastereoisomers for each amino acid. Furthermore, in comparison with peptides composed only of α-amino acids (α-peptides), β-peptides show higher resistance to enzymatic hydrolysis [34] and provide very stable helical secondary structures with very short peptide sequences [33]. For this reason, they self-assemble into different complex structures of controlled geometries [35,36], making them an extraordinary template for the design of novel materials [37,38]. Furthermore, β-peptides have been demonstrated to efficiently mediate the electron transfer process [39,40], expanding their potential application also in the field of energy and bioelectronic materials.

**Figure 2:**
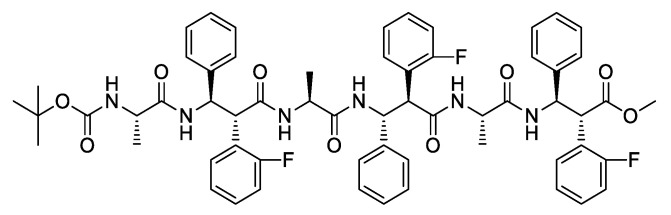
Chemical structure of the *NH*-Boc-(*S*-Ala-β-2*R*,3*R*-Fpg)_3_-OMe α,β-foldamer **1**.

Recently, we reported on a diastereoselective synthesis of a new class of β-amino acids, *syn*-S*,S*-β^2,3^-diaryl amino acids (β-2S*,3S*-Fpgs), differently substituted with aromatic groups [41]. In a previous study, the fluorine-substituted β-2*S*,3*S*-Fpg in combination with *S*-Ala [42] or *S*-Arg-*S*-Ala [43] were used to generate different supramolecular architectures, like nanotube or cationic nanospheres. More recently, a conformational study reported on tetra- and hexapeptides of general formula NH-Boc-(*S*-Ala-β-2*R*,3*R*-Fpg)_n_-OMe and NH-Boc-(*S*-Ala-β-2*S*,3*S*-Fpg)_n_-OMe was reported [44]. In particular, we demonstrated that the hexapeptide containing the α-amino acid *S*-Ala and the β-amino acid β-2*R*,3*R*-Fpg, that is the peptide NH-Boc-(*S*-Ala-β-2*R*,3*R*-Fpg)_3_-OMe (denoted in the following as **1**, Figure 2) gives rise to a very stable extended β-strand conformation. Moreover, NMR studies demonstrated that, when dissolved in CDCl_3_, it could also self-assemble into antiparallel β-sheet structures, stabilized by intermolecular H-bonds and π-π interactions [44]. This result is very important since the literature contains only few examples of α,β-repeating sequences containing acyclic amino acids and most of them give rise to helix constructs [45,46,47,48,49].

The aim of this work is the study the self-assembly capability of peptide **1**. This has been performed in solution by absorbance and fluorescence measurements, and by AFM and fluorescence microscopy on mica surfaces. Molecular dynamics (MD) simulations have been performed to investigate the structure at the atomistic level. We have also performed electronic conductivity measurements of this peculiar supramolecular assembly, discovering that it is in line with the value reported for other peptide β-sheet assemblies [50].

## 2. Materials and Methods

### 2.1. Molecular Modelling

Molecular mechanics parameters for the non-natural β-2*R*, 3*R*-Fpg amino acids, as well as the OMe protecting group, were obtained as described elsewhere [44]. The ff14SB force field was used in all simulations [51]. The MD simulations and trajectory analyses were performed with the Amber18, Amber20 and AmberTools21 packages [52]. The CUDA accelerated version of the pmemd and cpptraj modules was used to run MDs and trajectory analysis, respectively [53,54].

In the peptide model **1**, an acetyl group was used instead of BOC for computational efficiency. The simulation protocol to obtain the final 6 × 8 assembly was made by three separate steps. The first step was designed to explore the nucleation phase leading to one or more interactions involving different units of the peptide **1**. Thus, a system consisting of 12 randomly placed peptides **1**, initially constructed in an extended configuration, was created by using the Packmol software [55]. The system was solvated by a cubic box, extending up to 10 Å from the solute, filled by 9298 CHCl_3_ molecules. The system was equilibrated to the final temperature of 300 K through several MD runs at constant number of molecules, volume, and temperature (NVT) and constant number of molecules, pressure and temperature (NPT), using a protocol described elsewhere [44]. A classical MD (cMD) simulation of 50 ns was then done in NPT conditions to retrieve the boost parameters for potential and dihedral energy to be used for aMD. The system was made by 96 solute residues and 48,062 atoms. The average potential (EPTOT) and dihedral (DHIED) energy were −48,383.9 and 695.0 kcal/mol, respectively. The following setup was chosen for the 1 μs aMD NPT input: iamd = 3, ethreshd = 1031.0, alphad = 67.2, ethreshp = −40,694.0, alphap = 7689.9. Since we were interested in the dynamics of bonds involving hydrogens, the SHAKE algorithm was not used and a timestep of 1 fs was chosen as suggested elsewhere [56]. All the other parameters were left as default or as described elsewhere [44].

The second step was designed to simulate the association of β-sheet dimers. The starting geometry was constructed by randomly placing two peptide **1** antiparallel β-sheet dimers, whose geometry was retrieved by the aMD trajectory of step 1, into a periodic box filled with 834 CHCl_3_ molecules. The same protocol described above was adopted for equilibration, preliminary 50 ns of NPT cMD and 1 μs of NPT aMD simulation. The following parameters were used/modified in the aMD input: average EPTOT = −3381.9; average DHIED = 235.35; number of solute residues: 32; total number of atoms: 3759; ethreshd = 347.35, alphad = 22.4, ethreshp = −2780.5, alphap = 601.4.

The third step was designed to evaluate the stability of the final structure consisting of six layers, each one composed by eight peptides **1** forming an antiparallel β-sheet. The starting structure was manually generated by using the antiparallel β-sheet tetramer, obtained in the previous step, as a building unit. The 6 × 8 assembly was then solvated by a cubic CHCl_3_ box extending up to 15 Å from the solute (a total of 5110 solvent residues were added). The system was equilibrated up to 300 K by multiple MD runs at both NVT and NPT for a total of 1.6 ns. A 1 μs NPT cMD run was then conducted using a Langevin thermostat with a collision frequency of 2.0 ps^−1^. The PME algorithm, was used to treat long-range electrostatic interactions with a cut off for of 8.0 Å, the Berendsen barostat was used to keep pressure at standard conditions and the SHAKE algorithm was applied to constrain covalent bonds involving hydrogens.

### 2.2. Materials

Spectrograde solvents (Carlo Erba) were exclusively used. Water was distilled and passed through a Milli-Q purification system.

Peptide synthesis. The peptide **1** has been synthesized by us, and its synthesis and complete characterization is already present in literature [44].

Interdigitated electrodes. Interdigitated Concentric Gold Electrodes were bought from Metrohm DropSense (Herisau, Switzerland). Gold interdigitated concentric electrodes are made on a glass substrate, with bands/gaps of 10 µm. Electrodes were rinsed with ethanol and dried under a gentle argon flow before the peptide deposition.

Mica surfaces. 100 × 100 mm mica sheets of 0.15 mm thickness were bought from Goodfellow Cambridge Limited (Huntingdon, UK) and they were rinsed with ethanol and dried under a gentle argon flow before the peptide deposition.

### 2.3. Methods

UV-Visible Absorption. Absorption measurements were carried out on a Cary 100 SCAN (Varian, Palo Alto, CA, USA) spectrophotometer. All experiments were carried out in quartz cells of variable optical lengths (0.1, 0.5 and 1.0 cm) at millimolar concentrations.

Steady-state Fluorescence. Steady-state fluorescence experiments were carried out on a Fluoromax-4 spectrofluorimeter (Jobin-Yvon, Longjumeau, France) operating in the single-photon counting (SPC) mode. For the fluorescence measurements, the peptide **1** was dissolved in CH_2_Cl_2_ at a concentration of 2.8 × 10^−3^ M and the solution was inserted into a 1 cm × 0.4 cm asymmetric quartz cuvette, to reduce the inner filter effect. The fluorescence emission spectrum of the filaments was obtained by exciting the sample at 315 nm, while the “standard” spectrum of the aromatic groups was obtained by exciting the sample at 260 nm.

Time resolved fluorescence. Time-resolved fluorescence measurements were carried out on an EAI Life-Spec PS equipment (Edinburgh Analytical Instruments, Edinburgh, UK), operating in the single photon counting (SPC) mode. Excitation was achieved by a diode nanoled by Hamamatsu Photonics operating at λ = 298 nm and keeping the emission at 385 nm. A cut-off filter (λ = 305 nm) was used to minimize contamination from the scattered light. The temperature was controlled at 25 ± 0.1 °C with a thermostated cuvette holder. Experimental decays were fitted through iterative deconvolution of discrete exponential functions or continuous lifetime distributions by using standard software licensed by Edinburgh Analytical Instruments.

AFM measurements. Atomic force microscopy measurements on peptide films dried on a mica surface were performed in air using a Veeco Multiprobe IIIa (Santa Barbara, CA, USA) instrument. Experiments were carried out at room temperature (20 °C) in tapping mode by using NanoSensors Si tips with a force constant of about 40 N m^−1^ and a typical curvature radius on the tip of 7 nm.

Microscopy measurements. Fluorescence microscopy measurements have been performed with an Axio Scope microscope (Carl Zeiss MicroImaging Gmbh, Oberkochen, Germany) equipped with a CCD AxioCam ICm1 camera and a mercury lamp HBO 50.

Electrochemistry. The conductive properties of the fibrils were investigated by two-terminal transport experiments performed onto interdigitated electrodes. The fibrils were deposited on the electrode structures by cast deposition of a 10 μl drop of the 2.8 mM fibrils suspension of **1** in CH_2_Cl_2_.

The sample was then maintained overnight at ambient conditions. Such a procedure resulted in efficient fibrils deposition across the electrodes. The presence of peptide **1** fibrils between electrodes was assessed by optical microscopy analyses.

The devices for electronic transport experiments consisted of inter-digitated concentric gold electrodes fabricated on a glass substrate, with bands/gaps of 10 *µ*m. Electrodes were rinsed with ethanol and dried under a gentle argon flow before the peptide deposition.

Control experiments carried out on empty devices (i.e., without fibrils) detected very low-current signals (always <1 pA). The sample current was measured by means of a PG 310 potentiostat (Heka Elektronik, Lambrecht, Germany) at ambient conditions (20–25 °C, atmospheric pressure, 50–60% humidity).

## 3. Results

### 3.1. Spectrocopic Measurements

**Figure 3:**
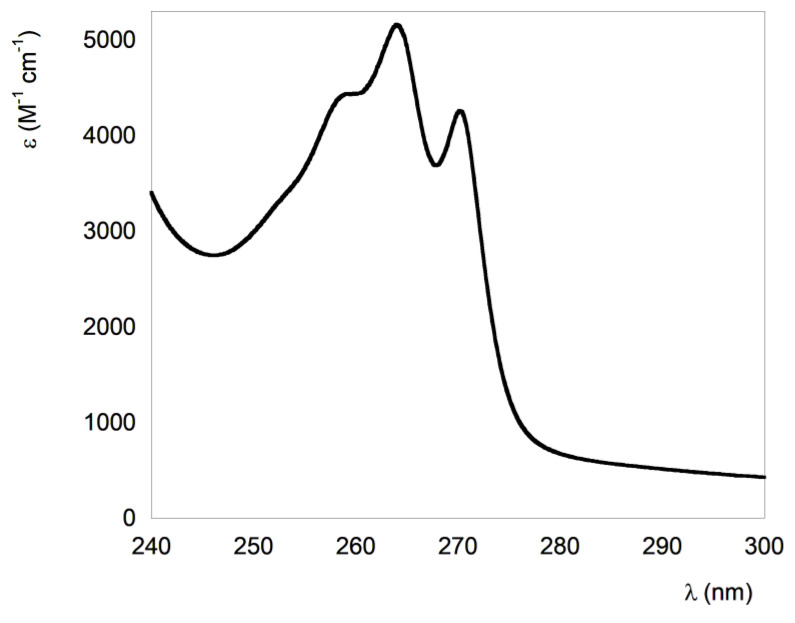
UV spectrum of **1** in CH_2_Cl_2_ solution.

The aromatic groups of the peptide (fluorobenzene and phenylalanine) absorb in the UV range, with an absorption maximum at 263 nm (Figure 3). The absorption spectrum of **1** in CH_2_Cl_2_ solution shows a band typical of the symmetry forbidden π → π* transition of the phenyl groups. The molar extinction coefficient for peptide **1** was found to be 5160 ± 300 M^−1^ cm^−1^. Since the fluorobenzene molar extinction coefficient at 263 nm is 1250 M^−1^ cm^−1^, and the phenylalanine molar extinction coefficient at 263 nm is 143 M^−1^ cm^−1^, the observed value is higher than the value obtained by the sum of three fluorobenzene and three phenylalanine groups (theoretical molar extinction coefficient at 263 nm = 4180 M^−1^ cm^−1^), suggesting interaction at the fundamental state between the aromatic groups [57].

This result is in agreement with the β-sheet assembly found in molecular dynamics simulations and experimentally from NMR studies [44], suggesting close interactions between aromatic groups. Furthermore, we verified that after one month, the baseline improved, suggesting that the aggregation has a slow kinetic.

**Figure 4:**
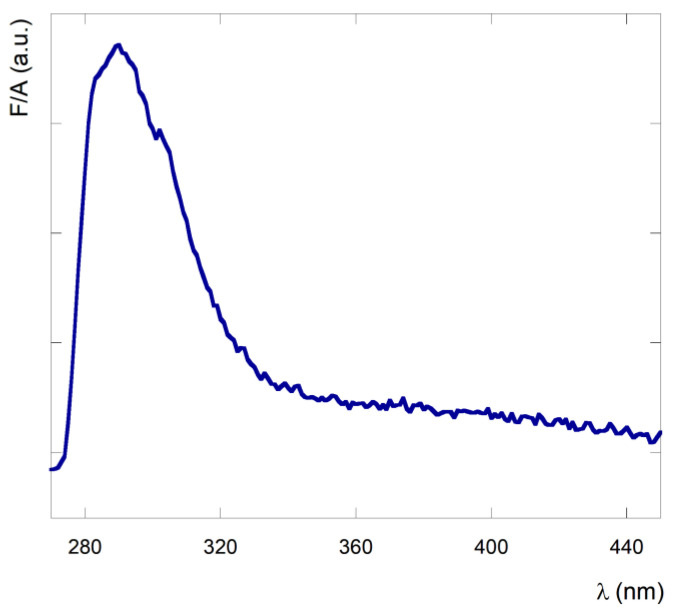
Fluorescence spectrum of peptide **1** in CH_2_Cl_2_ solution at a concentration of 2.8 × 10^−3^ M. λ_ex_ = 260 nm.

The canonical fluorescence spectrum performed in the UV emission region of the peptide, by exciting the sample at 260 nm is reported in Figure 4. The peptide exhibits the typical emission band of the phenyl fluorophore, with a maximum at 290 nm and a quantum yield of 0.05 ± 0.02.

Furthermore, the increase of the baseline signal in the fluorescence emission spectrum is in agreement with the scattering caused by the peptide aggregation.

**Figure 5:**
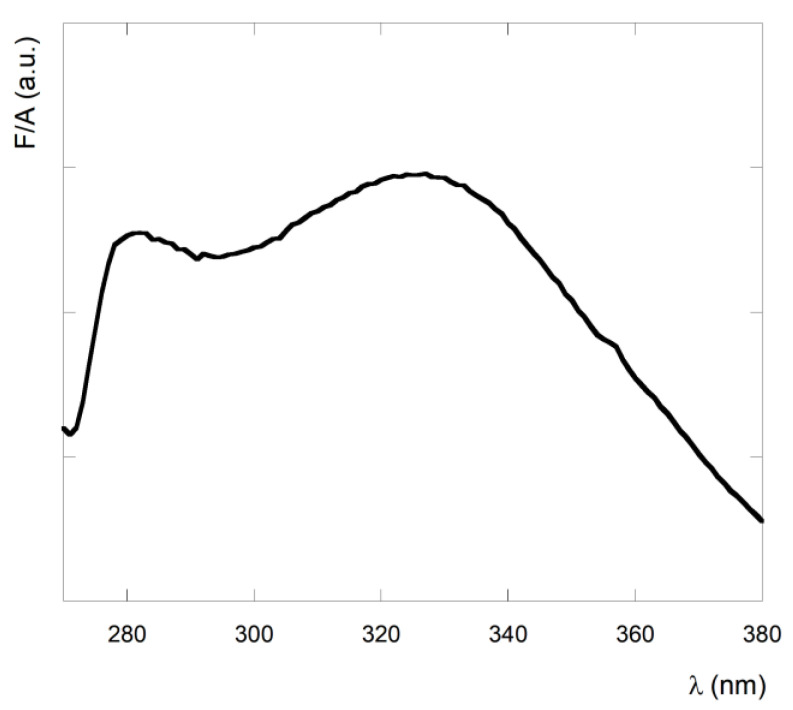
Fluorescence spectrum of peptide **1** fibrillar nanostructures in CH_2_Cl_2_ solution at a concentration of 2.8 × 10^−3^ M. According to Ref. [61] the fluorescence emission spectrum of the filaments was obtained by exciting the sample at λ_ex_ = 315 nm. Continuous line: fresh solution, dotted line: solution after ten days.

Fluorescence measurements of **1**-aggregate in CH_2_Cl_2_ solution upon near UV/blue excitation (λ_ex_ = 315 nm) showed the typical spectrum of the fibrillar nanostructures in the visible range [58,59,60,61,62] (Figure 5), with an emission maximum at 385 nm. The origin of this spectrum is much debated in the literature, but most of the papers agree that this signal is typical of fibrillar nanostructures [58,59,60,61,62]. In particular, Kaminski and collaborators reported that this measurement could be a good alternative to the assay performed with Thioflavin T [61].

**Figure 6:**
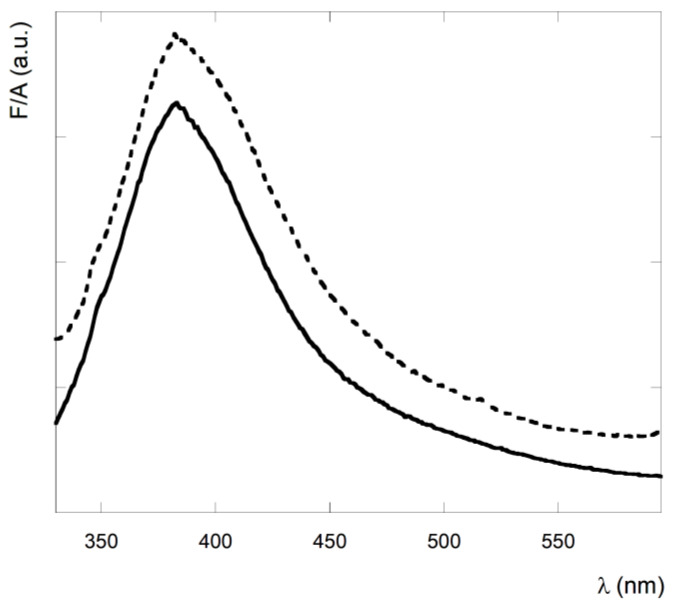
Fluorescence excitation spectrum of peptide **1** in CH_2_Cl_2_ solution at a concentration of 2.8 × 10^−3^ M. λ_em_ = 400 nm.

As a matter of fact, the excitation spectrum of this solution obtained at λ_em_ = 400 nm, showed two bands: one at 275 nm and a second at 330 nm (Figure 6). This last signal improves over time, suggesting a correlation between the aggregation kinetic and this fluorescence indicator (Supplementary Figure S1). Furthermore, our results show that the emission intensity with maximum at 385 nm (Figure 5, dotted line) and the baseline of the fluorescence spectrum with a maximum at 290 nm (Supplementary Figure S2) also increase over time, confirming their correlation with the aggregation process.

### 3.2. Morphology of the Supramolecular Assembly

**Figure 7:**
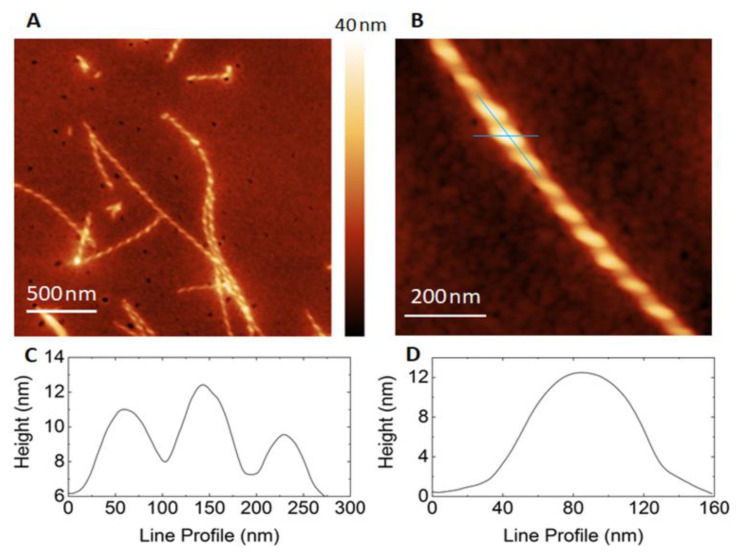
AFM characterization of peptide **1** supramolecular structure, drop-casted onto mica surface. In (**A**,**B**), representative AFM images of the fibrillar structure on the mica surface are shown. Cross sections through the structure are shown below: (**C**) corresponds to the line profile along the fibril structure (reported in (**B**) as a vertical blue line); (**D**) corresponds to the horizontal line profile (reported in (**B**)).

Inspection of the peptide supramolecular structure obtained on a mica surface by AFM measurements revealed a unique and highly ordered morphology (Figure 7A). At first sight, these images showed the formation of elongated fibres. These structures were obtained after cast deposition and overnight solvent evaporation on the mica surface of a 10-μL-drop of a 1 mM solution of **1** in CH_2_Cl_2_. A deep analysis of the image showed that the long fibres have an unexpected and fascinating “rope” shape, exhibiting higher and lower areas, systematically repeating over its length. The vertical height profile along the rope-shape nanofibers (Figure 7C) exhibits a minimum height of 2 nm, consistent with the length of the elongated peptide, and a maximum height of 5–7 nm. The horizontal profile (Figure 7D) showed a width of 100 nm line profile reported in Figure 7B. We performed all AFM measurements at room temperature in tapping mode.

### 3.3. Molecular Organization within the Assembly

**Figure 8:**
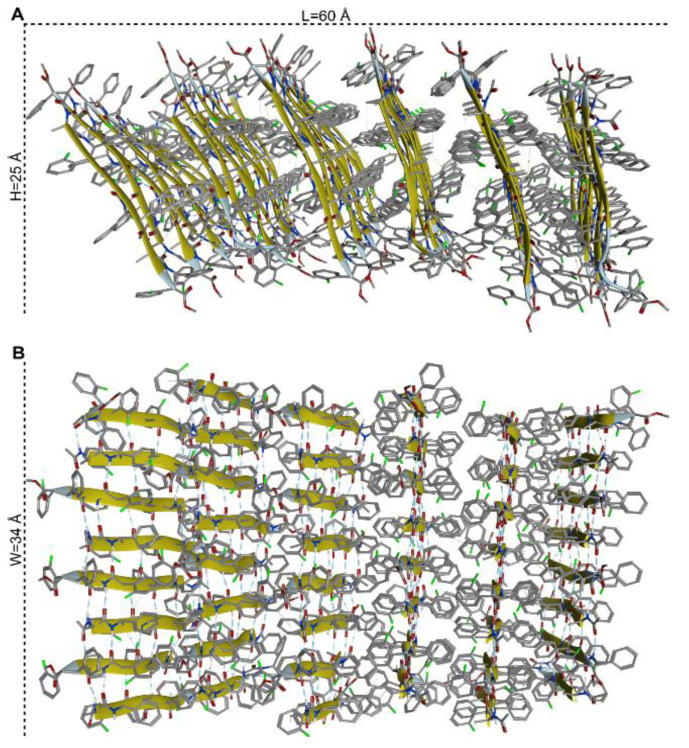
Side (**A**) and top (**B**) views of the 6 × 8 assembly of model peptide **1**. The geometry corresponds to the representative structure of the most populated cluster (population = 85%) obtained by cluster analysis of the last 400 ns of the 1 μs MD trajectory in explicit solvent. L is the average distance between the C-terminus of corresponding peptides in the first and the last β-sheet; W is the average distance between the C=O groups at the C- and N-terminal protections of the first and the last peptide within each β-sheet, while H corresponds to the average length of each peptide, as measured between the C=O groups.

The molecular self-assembly was analysed at the atomistic level by molecular dynamics (MD) simulations. The first stage of the process was modelled by accelerated MD (aMD). This enhanced sampling technique can simulate chemical events occurring at a ms time scale, but within only a few hundreds of ns of simulation time [63,64,65,66,67,68]. The system was built by randomly placing 12 units of peptide model **1** into a periodic box filled with CHCl_3_ at a concentration of approximately 15 mM. This choice represented a reasonable compromise between experimental conditions, computational cost and the availability of solvent parameters within the Amber software package [52]. Within 1 μS of aMD, by monitoring the number of intermolecular H-bonds vs. time (Supplementary Figure S4), we observed the formation of two nearly identical antiparallel β-sheet dimers (Supplementary Figure S5). A second model was then generated to explore the dimer–dimer association. The two antiparallel dimers obtained in the previous step, whose geometries were retrieved from the last frame of the aMD trajectory, were placed randomly into a CHCl_3_ box. A final concentration of 60 mM was chosen to both limit the computational cost and simulate a higher concentration, as it occurs during solvent evaporation. Again, within 1 μS of aMD simulation time, we observed the formation of an antiparallel β-sheet tetramer (Supplementary Figures S6 and S7). Starting from this geometry, we built an assembly made by six β-sheets of eight peptide each that was solvated by CHCl_3_ at a concentration of about 110 mM and subjected to 1 μs of classical MD simulation. The 6 × 8 assembly was rather stable during the whole simulation (Supplementary Figures S8 and S9), and the principal structure obtained by cluster analysis of the last 400 ns of MD trajectory (main cluster population = 85%) is shown in Figure 8. Results of the computational study suggest that foldamer **1** can self-assemble driven by two different forces: first, the peptide in an extended conformation can self-assemble through H-bonds giving rise to an antiparallel β-sheet; afterward the π-π interactions between the lateral aromatic groups can give rise to many peptide β-sheet layers (Figure 8).

Theoretically, the assembly could be elongated on both the L-(horizontal) and W-(vertical) dimension, as described in Figure 8A and 8B, respectively. The W-elongation can be obtained by adding strands to the antiparallel β-sheet through H-bonds. Conversely, the L-elongation can occur by the addition of further β-sheet layers to the assembly through hydrophobic interactions. The aryl–aryl interaction between adjacent β-sheet layers was verified by monitoring the distances between the centroids of aryl groups of selected β-2*R*,3*R*-Fpg residues (Supplementary Figure S10). For all the considered interactions, the average distances were found to be between 5.2 and 6.3 Å and were stable over the whole 1 μs MD simulation. These values are well below the limits reported for π-π stacking in proteins [69], confirming the role of these interactions in driving the L-elongation of the assembly. According to the model in Figure 8, the assembly appears to be rather flat along the W-dimension, where the elongation is mediated by H-bonds. Conversely, some curvature can be observed along L (Figure 8A and Figure S10), where hydrophobic interactions are responsible for the elongation. Accordingly, we expect that the system presents higher flexibility in the L-dimension, since the π-π interactions between the aryl groups are weaker compared to H-bonds (but covering greater distances due to their long-range nature). Conversely, this structure should be relatively rigid in the W-dimension since H-bonds are stronger interactions. In general, amyloid fibrils are formed by the self-assembly of beta-sheet layers, brought together by aryl–aryl interaction between adjacent β-sheet layers [70,71]. Within each sheet, every segment is bound to its two neighbouring segments through stacks of both backbone and side-chain hydrogen bonds. The structure shows the stability of amyloid fibrils, their self-seeding characteristic and their tendency to form polymorphic structures.

**Figure 9:**
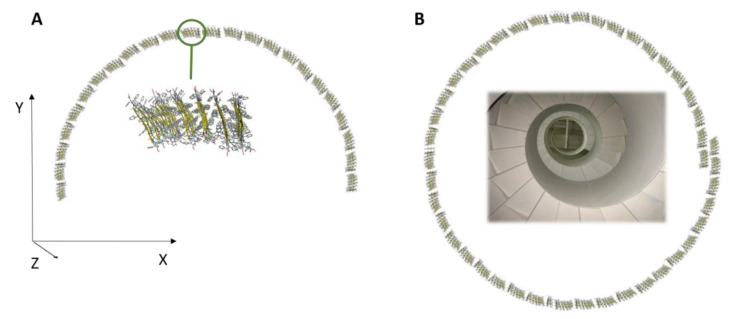
Views of the assembly of the 6 × 8 repetition of model peptide **1**, following the system curvature. (**A**) View of the arc. (**B**) View of the folding of the arc along the Z-axis, like a spiral staircase (inside the structure).

These structures reveal the details of the packing interactions by which the constituent β-strands are assembled hierarchically into protofilaments, filaments and mature fibrils, which often show chirality in the self-assembly process. Since our eight-peptide β-sheet model composed of six layers measures about 60 Å in length and it also shows a curvature (Figure 8A), we can speculate that the self-assembly of 29 repeating unit of it along the X dimension, can give rise to an arc (Figure 9A), the base of which is 120 nm long. This dimension corresponds to the width of the rope-shape filament (Figure 7D). Continuing the self-assembly along the Y-dimension and along the Z-dimension, a rope-shape filament can be obtained, like it happens in a spiral staircase (Figure 9B).

### 3.4. Electronic Conduction Measurements

**Figure 10:**
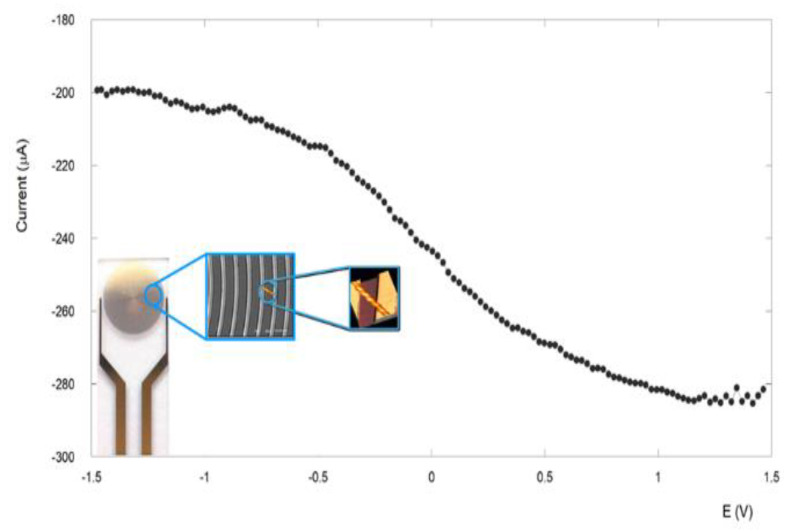
Typical current-voltage features of the nanofibril ropes. Inset: schematic representation of a peptide fibril across the interdigitated gold electrode. The dimension of bands/gaps of this electrode is 10 μm, while the diameter of the gold disk is 5 mm.

The conductive properties of these filaments were investigated by a two-terminal transport experiment carried out at ambient conditions. The device for these measurements consisted of interdigitated electrodes with gaps of 10 μm. A representative image of this device obtained with an optical microscope is reported in Supplementary Figure S12. The fibrils were deposited onto the electrodes by cast deposition of a 10-μL drop of a 2.8 × 10^−3^ M fibril suspension in CH_2_Cl_2_. After solvent evaporation, several fibrils were seen to span the interelectrode gap. With a −1.5-V and +1.5-V potential application, a high current was detected, typically in the range of 48–50 μA (Figure 10) [56].

Control experiments carried out on empty devices (without peptide) revealed very low current signals (less than pA). The conductivity of the immobilized fibres has been found to be 32 mS/cm at an applied potential of 1.5 V. This value is in line with those reported in other peptide systems [72,73,74,75], demonstrating the good conduction capabilities of this supramolecular rope. A possible explanation of this remarkable conductivity relays on the outstanding self-assembling capabilities of the α,β-peptide **1**. As a matter of fact, electronic conduction across peptides depends on how regularly they self-assemble into an ordered supramolecular structure and their consequent capability to give rise to delocalization. To better understand this concept, a first distinction between the process of electron transfer and electron transport should be pointed out [76]. In general, electron transfer is the exchange of an electron that occurs as a redox event between an ionically conductive electrolyte and a peptide in contact with the electrolyte [69,70,71,72,73,74,75,76]. On the contrary, short-range electron transport (ETp) refers to the electron flow through a peptide in the absence of the electrolyte [77,78].

Etp therefore requires electron flow across a peptide between two electronically conducting electrodes without a charge-screening electrolyte, like in our experiment. In general, the ET process is widely studied and rationalized based on theoretical models, while Etp is much less so. Recently, it was demonstrated that the models used to rationalize ET may be applied also to Etp through peptides and proteins [79]. These models can be extended to the interpretation of transport across longer distances through supramolecular architectures of peptide building blocks, interpreting long range ET as a series of sequential short range ET events and long range Etp as continuous electron flow through degenerate electronic states, in which the states can take the form of electronic bands. In general, band formation occurs in highly periodic or crystalline organic materials. In peptides the periodicity produces band gaps that vary from the semiconducting value (Eg ≤ 4 eV) to insulating one (Eg ≥ 4 eV) [72,73,74,75,76,77,78,79,80]. Interestingly, this value can be decreased by incorporating aromatic residues in the structure, if they promote delocalization through π-stacking, that is π-π orbital interactions. These interactions support electron transport across peptides if they have the long-range periodicity, which is necessary to realize delocalization along the length of the supramolecular structure and if the distance between aromatic residues is ≤3.4 Å, promoting delocalization between adjacent residues [50]. In general, natural aromatic amino acids exceed the 3.4 Å distance for efficient orbital delocalization, making these systems less efficient in long-range ETp. The non-coded β-2*R*,3*R*-Fpg reported in this work makes possible the delocalization between adjacent residues, since π-π-interactions between different β-2*R*,3*R*-Fpg act as an aromatic molecular zipper between the different β-sheet layers, contributing to the assembly and structural stability of the supramolecular architecture [81]. It has been demonstrated that assemblies exclusively stabilized by β-sheet hydrogen bonds have intermolecular distances (typically 4.7 Å) that preclude efficient orbital overlap (3.4 Å) [82,83]. In the present system, the short distance between aromatic groups (3.4 Å) in the supramolecular structure obtained by the self-assembly of the foldamer **1** favours delocalization and hence electron transport across the peptide, making this system more efficient than the others previously reported in the literature. Further, the dense network of hydrogen bonds can contribute on the good electron transfer properties of this system [49,84]. In conclusion, all these results clearly indicate that the α,β-peptide self-assembly properties make it possible to span the actual repertoire of peptide-based supramolecular structures, obtaining nanostructures with new and more performing properties than those already known, such as electron transport, which is very useful for the design of biocompatible bioelectronic devices.

## Data Availability

Not applicable.

## Supplementary Materials

The following supporting information can be downloaded at: https://www.mdpi.com/article/10.3390/nano13020333/s1, Figure S1: Fluorescence excitation spectrum of peptide 1 over time; Figure S2: Fluorescence spectrum of peptide 1 followed over time; Figure S3: Fluorescence-decay curve of the peptide 1 in CH2Cl2; Table S1: Fluorescence time-decay parameters; Figure S4: Graph showing the number of intramolecular H-bonds vs simulation time obtained from the analysis of the 1 μS trajectory of the first aMD run; Figure S5: Peptide structures; Figure S6: Graph showing the number of intramolecular H-bonds vs simulation time obtained from the analysis of the 1 μS trajectory of the second aMD run; Figure S7: Structure of the antiparallel β-sheet tetramer; Figure S8: Number of intramolecular H-bonds vs simulation time obtained from the analysis of the 1 μS trajectory of classical MD; Figure S9: RMSD vs simulation time obtained from the analysis of the 1 μS trajectory of classical MD simulation of the 6 x 8 peptide 1 assembly; Figure S10: Time evolution (1 μs of MD simulation) of representative distances between the centroids of the aryl groups of peptides belonging to adjacent β-sheet layers; Figure S11: AFM characterization of peptide 1 supramolecular rope structure; Figure S12: Optical microscopy image of the interdigitated electrode; Figure S13: Fluorescence microscopy image of the filaments of peptide **1.**
