# Supplementary material for: Non-Conventional Peptide Self-Assembly into a Conductive Supramolecular Rope
Source: Nanomaterials (Basel). 2023 Jan 13;13(2):333. doi: 10.3390/nano13020333 (PMC9867255; doi:10.3390/nano13020333)
Supplement: Supplementary file 1 [file nanomaterials-13-00333-s001.zip › nanomaterials-2150265-supplementary.pdf]

nanomaterials

Supporting Information of the paper: Non conventional peptide self-assemble into a highly conductive supramolecular rope

N. Forlano,[a] R. Bucci,*[b] A. Contini,[b] M. Venanzi,[a] E. Placidi,[c] M. L. Gelmi,[b] R. Lettieri,[a] E. Gatto,*[a]

[a] *Department of Chemical Science and Technologies, University of Rome Tor Vergata, Rome (Italy)*

[b] *Department of Pharmaceutical Sciences, University of Milan, Milan (Italy)*

[c] *Department of Physics, Sapienza University of Rome, Rome (Italy)*

Fluorescence measurements.

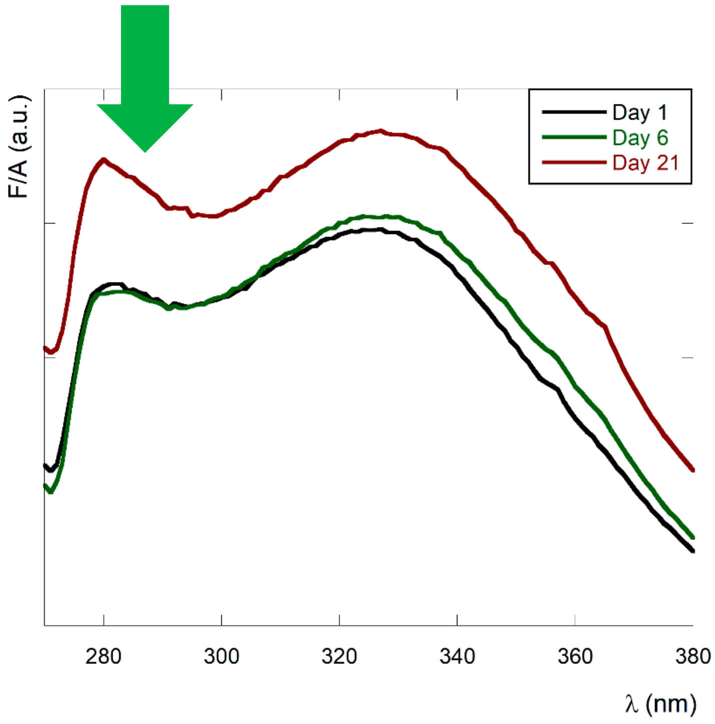

Figure S1. Fluorescence excitation spectrum of peptide **1** in CH_2Cl_2 solution at a concentration of $2.8 \cdot 10^{-3}$ M.. λ_{em}=400 nm, followed over time.

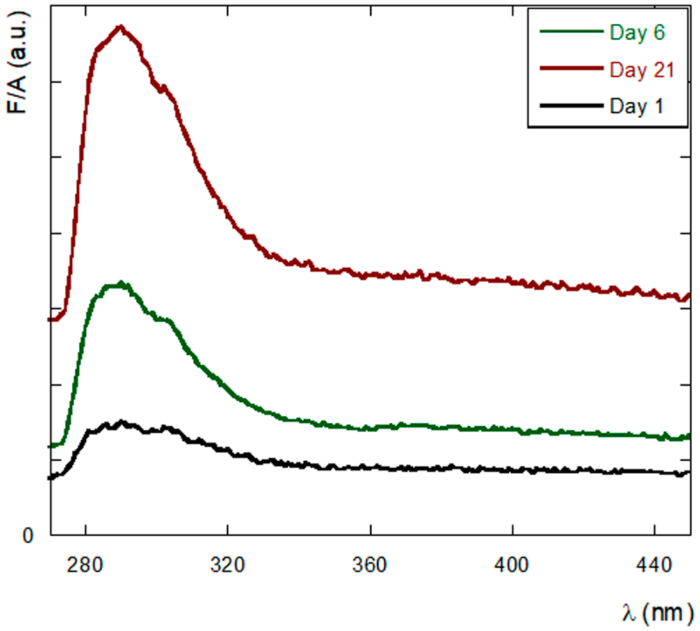

Figure S2. Fluorescence spectrum of peptide **1** in CH_2Cl_2 solution at a concentration of $2.8 \cdot 10^{-3}$ M.. λ_{ex}=260 nm, followed over time.

Time-resolved fluorescence measurements.

The decay profile of peptide **1** in CH_2Cl_2 (λ_{ex}= 298 nm; λ_{em}=385 nm) is show in Figure S3. Fluorescence time decay has been fitted through a multi-exponential analysis. In this kind of analysis, the time-decay of $I(t)$ vs. t is fitted by the following function:

$$I(t) = \sum_{i}^{n} \alpha_i \cdot e^{-\frac{t}{\tau_i}}$$

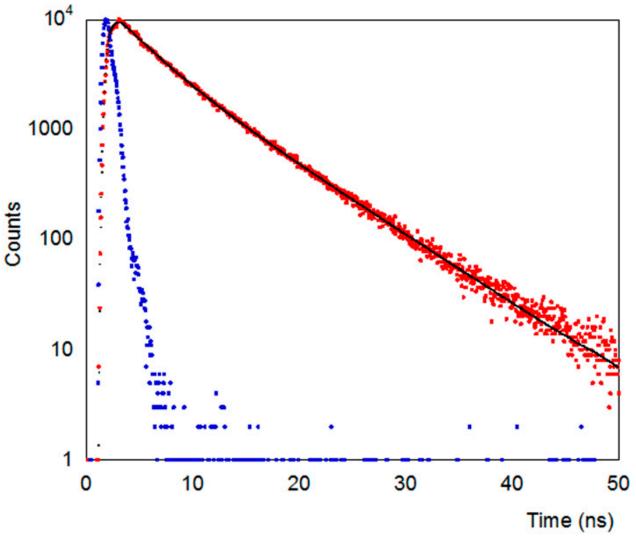

Figure S3. Fluorescence-decay curve of the peptide **1** in CH_2Cl_2 (λ_{ex}= 298 nm; λ_{em}=385 nm).

Time-resolved fluorescence measurements confirmed that the signal was due to fluorescence, having a lifetime in the order of several ns. Table 1 reports the value of the time-decay parameters of peptide **1** in CH_2Cl_2.

**Sample**	τ_1	α_1	τ_2	α_2	χ^2
Peptide **1**	0.72	0.37	3.63	0.63	1.3

Table S1. Fluorescence time-decay parameters (lifetimes (τ) and pre-exponents (α)) of excited peptide **1** in CH_2Cl_2, recovered by Multiexponential (ME) analysis (λ_{ex}= 298 nm; λ_{em}=385 nm).

Molecular modelling

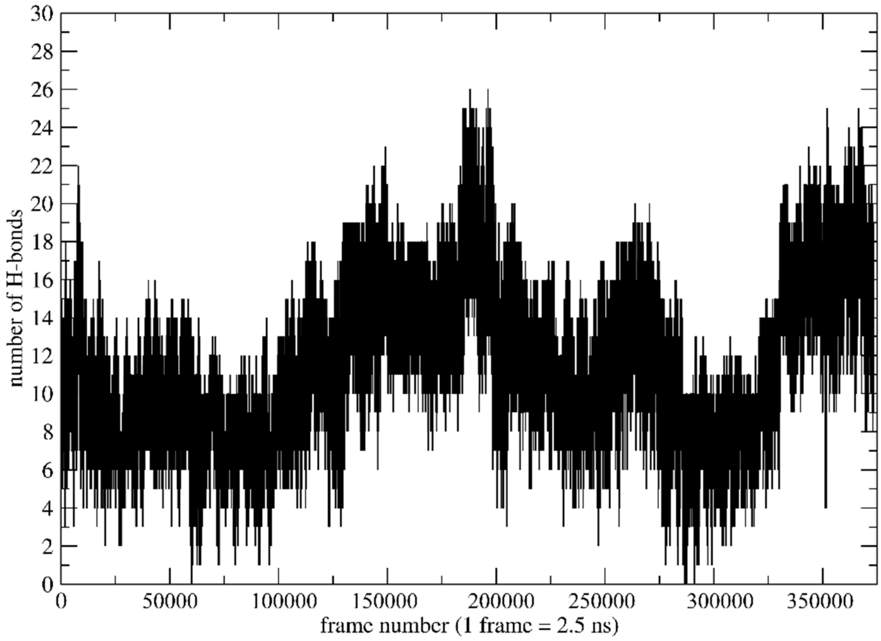

Figure S4. Number of intramolecular H-bonds vs simulation time obtained from the analysis of the 1 µS trajectory of the first aMD run. The simulation was done on a system of 12 units of peptide 1 solvated by $CHCl_3$ at a concentration of about 20 mM.

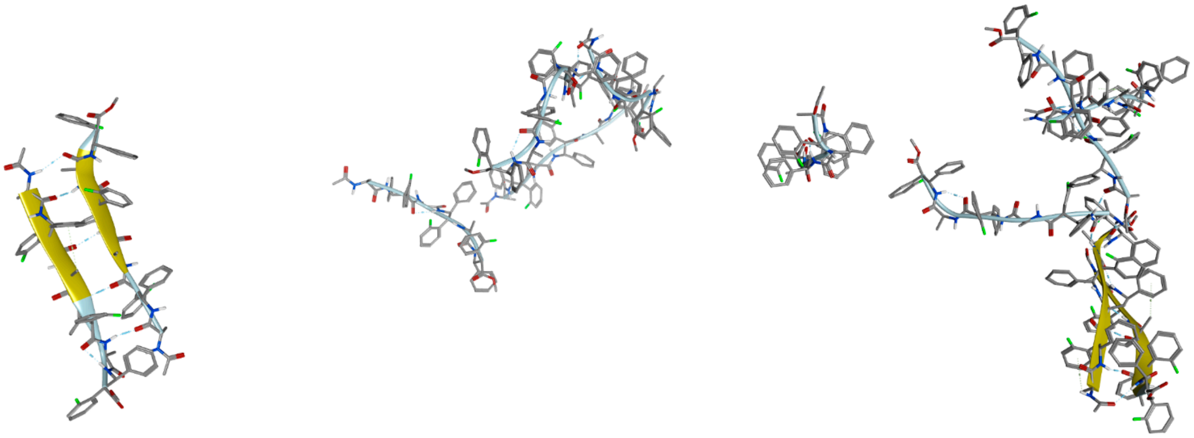

Figure S5. Structure of the last frame of the 1 μS aMD trajectory of 12 units of peptide **1** randomly placed in a box of explicit $CHCl_3$ (solvent not shown).

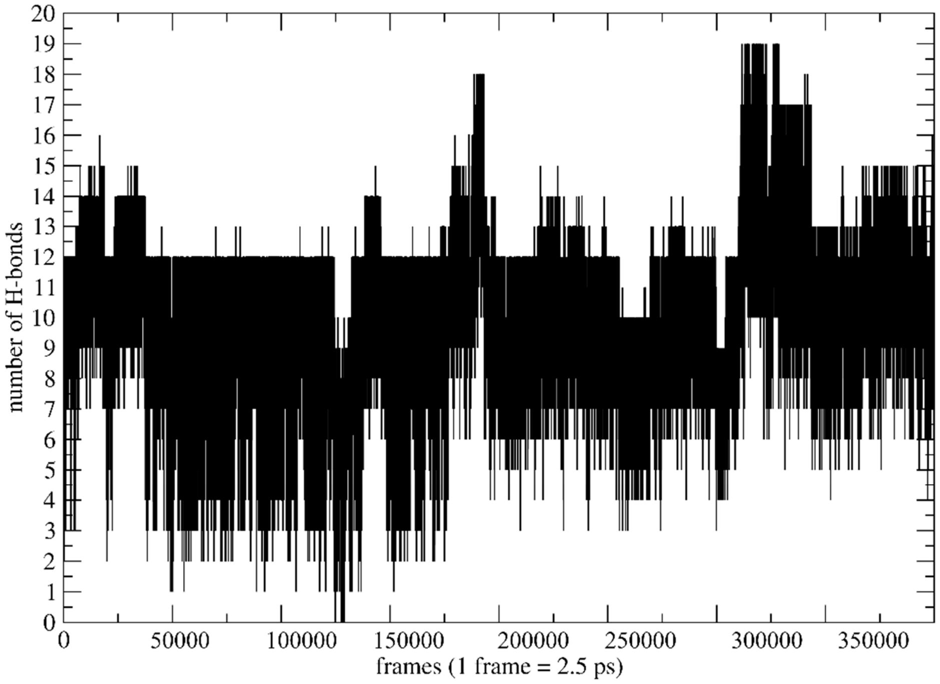

Figure S6. Number of intramolecular H-bonds vs simulation time obtained from the analysis of the 1 μS trajectory of the second aMD run. The simulation was done on 2 antiparallel β-sheets dimers of **1** peptide solvated by $CHCl_3$ at a concentration of about 60 mM.

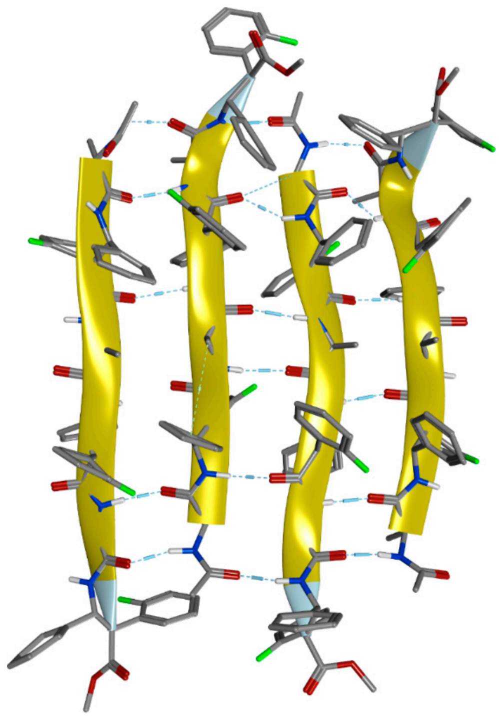

Figure S7. Structure of the antiparallel β-sheet tetramer. The geometry was retrieved from the frame number 320120 (approximately 800 ns of simulation time) of the 1 μS aMD trajectory of two antiparallel β-sheets dimers of peptide **1** randomly placed in a box of explicit $CHCl_3$ (solvent not shown).

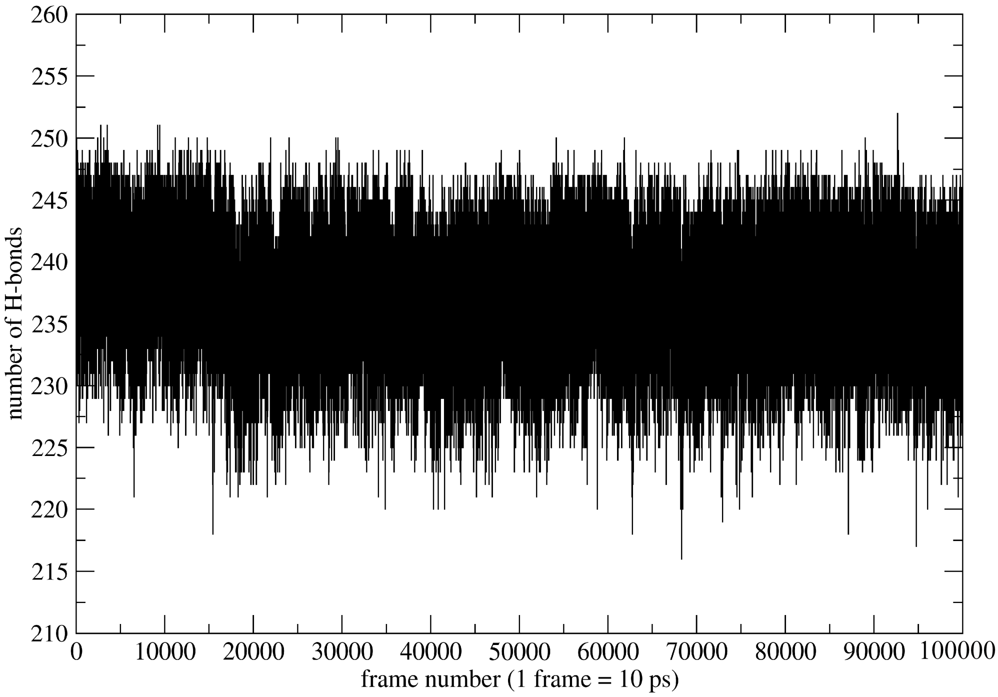

Figure S8. Number of intramolecular H-bonds vs simulation time obtained from the analysis of the 1 μS trajectory of classical MD. The simulation was done on an assembly of 6 β-sheets layers made by 8 units of peptide **1** each, in explicit $CHCl_3$.

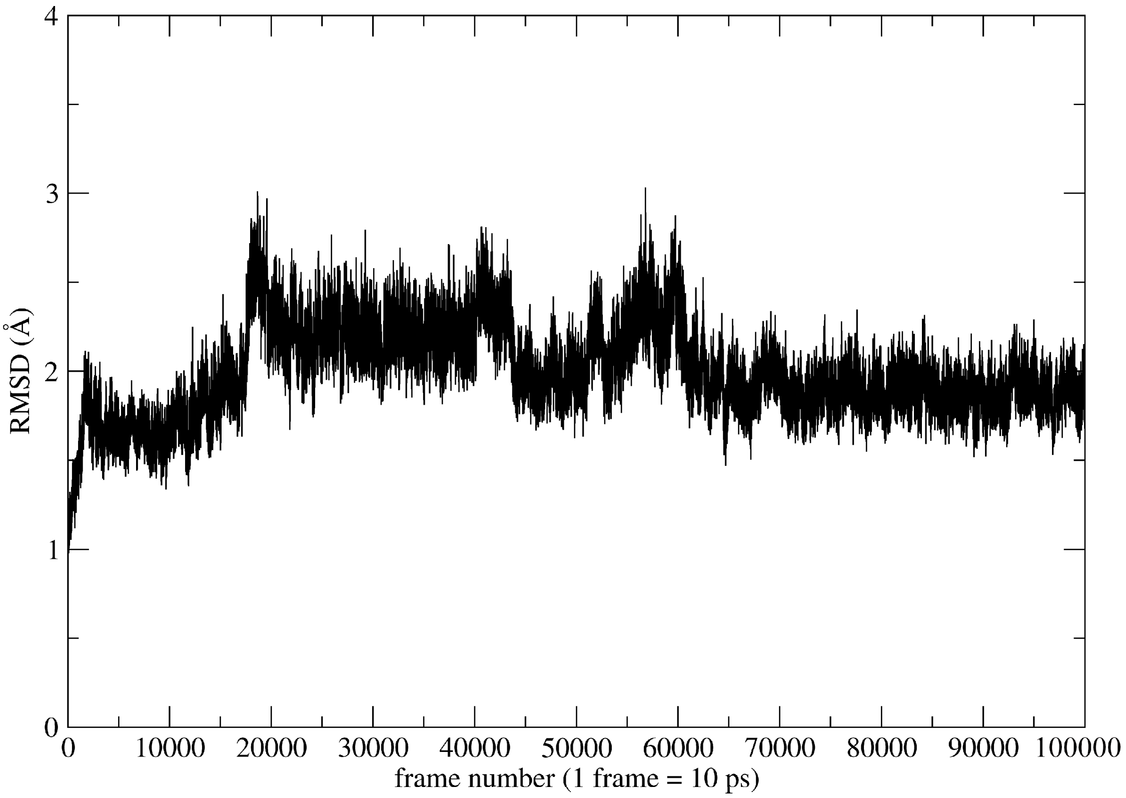

Figure S9. RMSD vs simulation time obtained from the analysis of the 1 μS trajectory of classical MD simulation of the 6 x 8 peptide **1** assembly.

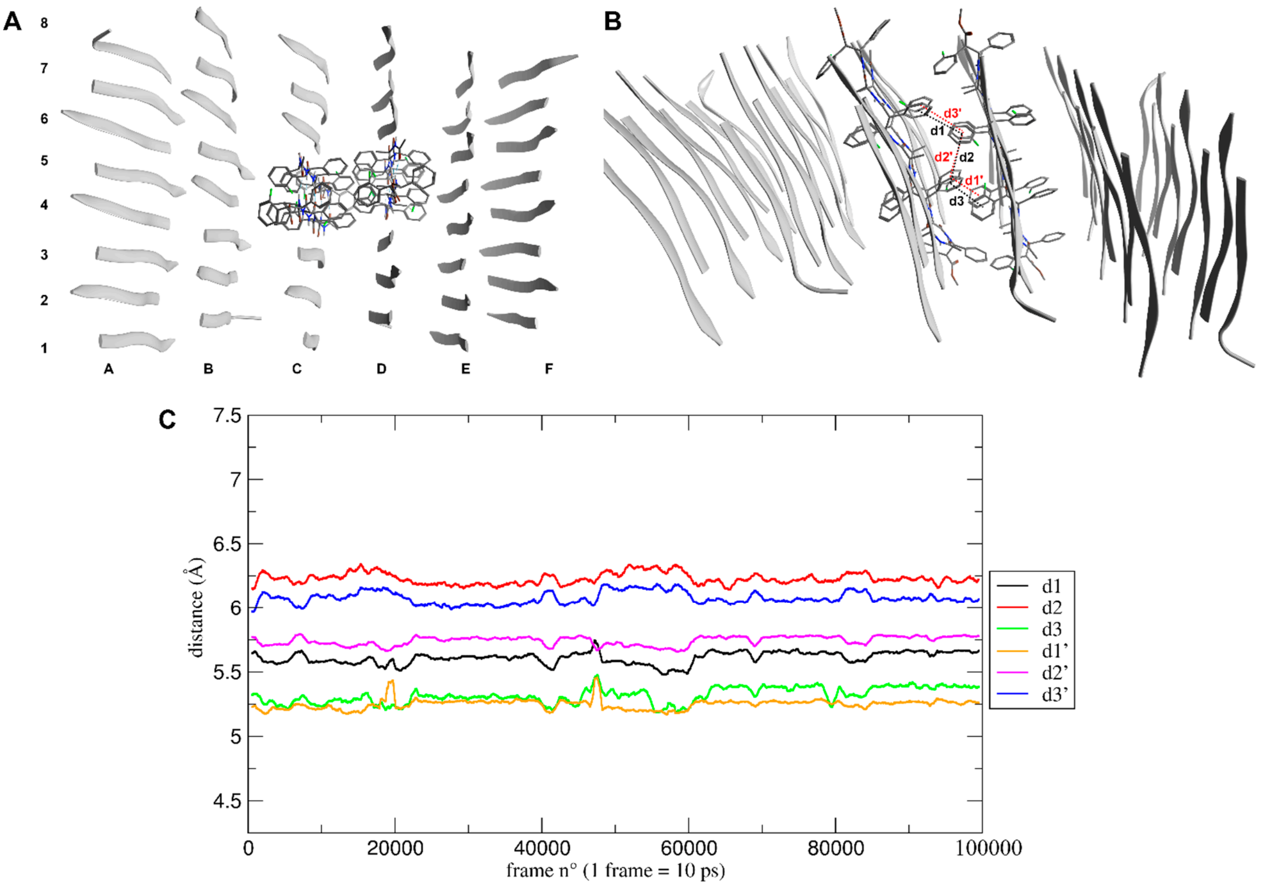

Figure S10. Time evolution (1 μs of MD simulation) of representative distances between the centroids of the aryl groups of peptides belonging to adjacent β-sheet layers. Panel **A** shows the top view of the supramolecular assembly with the coordinate system used to label peptides selected for the measurements herein reported. Distances were computed for the aryl-aryl interactions between the peptide pairs C4-D4 (d1-d3) and C5-D5 (d1′-d3′), chosen because of their central position in the 48mer assembly. Panel **B** shows the side view of the assembly and selected distances are highlighted; d1 represents the interaction between the β-2S,3S-Fpg residues in position 4 of peptides C4 and D4; d2 describes the interaction between β-2S,3S-Fpg in position 2 of C4 and β-2S,3S-Fpg in position 4 of D4; d3 represents the interaction between β-2S,3S-Fpg in position 2 of C4 and D4. Distances d1′-d3′ describe the same interactions but involving peptides C5 and D5.

AFM measurements.

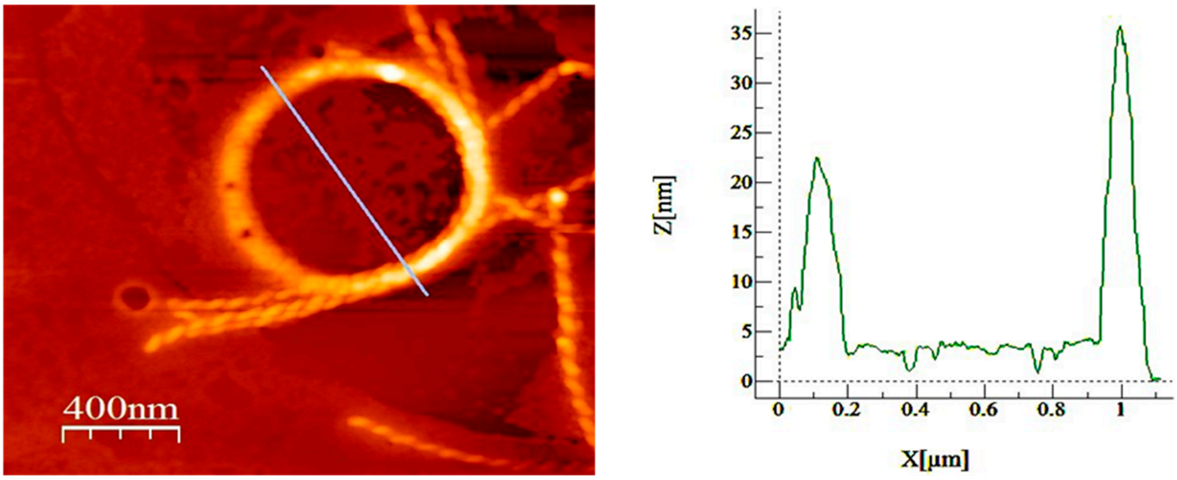

Figure S11. AFM characterization of peptide **1** supramolecular structure. Right: line profile reported in the AFM image on the left.

Fluorescence microscopy measurements.

Figure S12 shows the circular shape of the concentric interdigitated electrode, with have gaps of 10 µm. The image has been obtained with an optical microscope, using a 20x magnification.

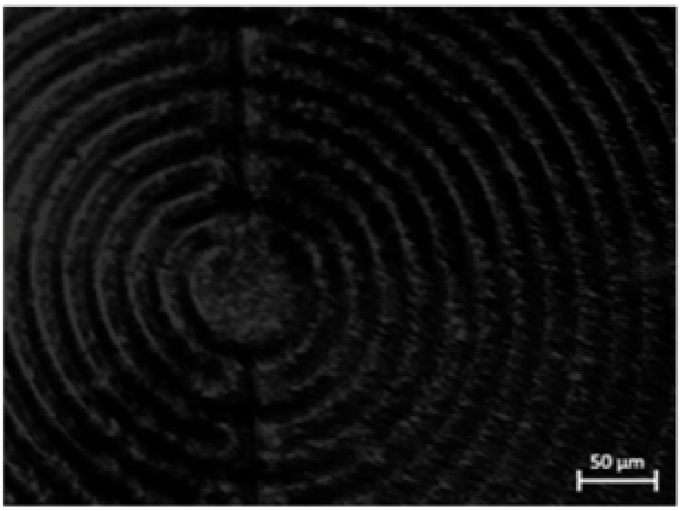

Figure S12. Image of the interdigitated electrode, obtained with a fluorescence optical microscope, applying a 20x magnification.

Figure S13 shows the fluorescent filaments of peptide **1** in the blue region. The fibrils were deposited onto the mica surface by cast deposition of a 10 µl drop of a 2.8 10^{-3} M fibril suspension in CH_2Cl_2.

After solvent evaporation, several fluorescent fibrils were seen, confirming that the emission is due to aggregation.

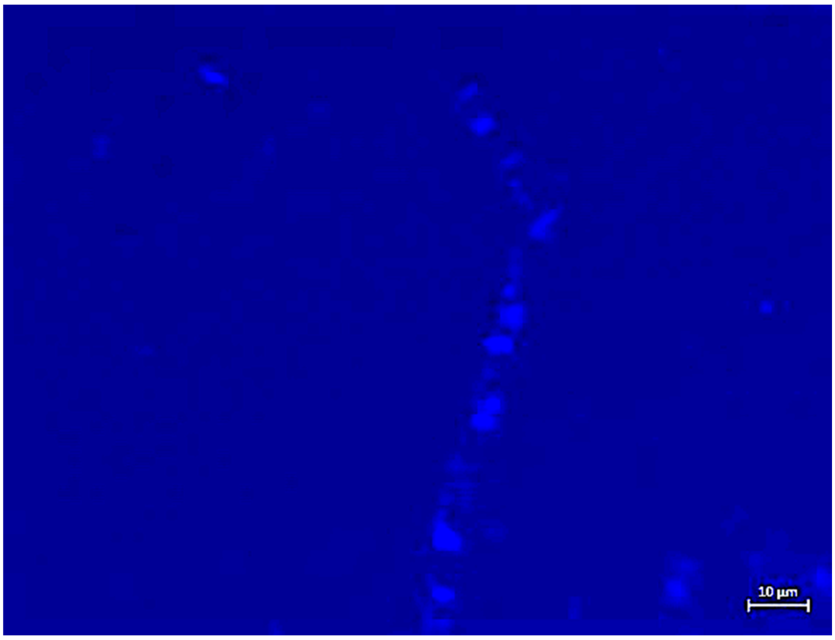

Figure S13. Image of the filaments of peptide **1**, obtained with a fluorescence optical microscope, using a 20x magnification in the DAPI region (blue channel).
